# The Cognitive Ability of Chinese Students with Dyslexia and Mathematical Learning Disabilities

**DOI:** 10.3390/children9121949

**Published:** 2022-12-12

**Authors:** Zhaoyu Li, Abdo Hasan AL-Qadri, Wei Zhao

**Affiliations:** 1 School of Education, Shaanxi Normal University; Xi’an 710062, China; 2 School of Humanities and Education, Xi’an Eurasia University; Xi’an 710065, China

**Keywords:** CHC theory, Chinese, dyslexia, mathematics learning disabilities

## Abstract

This study aims to investigate the core cognitive factors that affect reading and math performance of children of the grades 1–6 in Xi’an, Shaanxi Province, China, as well as the differences between children with dyslexia and mathematical disabilities (MD). Therefore, this study mainly evaluated the Cattell Horn Carroll (CHC) cognitive factors for 427 Chinese children and explored the core cognitive factors that affect Chinese children’s reading and math performance. Students with dyslexia (*n* = 34), students with mathematics learning disabilities (*n* = 34), and 34 normal children were randomly selected as the control group. In order to explore the differences in cognitive development, we analyzed the differences among the three groups (Dyslexia, mathematical learning disabilities (MD), and normal children). The results revealed the following: (1) almost all cognitive ability factors in this study are significantly related to students’ reading and mathematical achievements. (2) the core cognitive factors for predicting Chinese dyslexia students are crystallized intelligence, auditory processing and working memory. Executive function, spatial relationship and working memory are the core cognitive factors to predict Chinese children’s mathematical achievements. (3) in addition, there are differences in cognitive deficits between disabled Chinese children in reading and math, among which those with reading deficits have extensive auditory processing deficits; while children with mathematic deficits have worse executive function. Recommendations were made based on these findings.

## 1. Introduction

In previous studies, children with learning disabilities were screened mainly through three ways: the ability–achievement discrepancy approach, Response to Intervention (RTI) and cognitive and neuropsychological assessment of Processing Strengths and Weaknesses (PSW). However, a large number of subsequent studies believed that the ability–achievement discrepancy approach was inefficient to some extent [1], and the RTI was only a preventive method for early identification and intervention of students with learning disabilities, rather than a screening method for students with learning disabilities [2]. The PSW has significant advantages in accurately identifying students with special learning disabilities, understanding their learning problems, and providing targeted intervention plans [3]. Cognitive assessment plays a key role in identifying learning difficulties. Among the relevant human intelligence theories, Cattell Horn Carroll (CHC) cognitive ability theory provides a complete evidence-based theoretical framework for understanding the cognitive development of students with special learning disabilities by comprehensively describing human cognitive ability. Under the current research background, the research on the relationship between CHC cognitive ability and academic achievement has gradually increased, and more abundant evidence has proved that cognitive ability has different effects on academic achievement. The broad and narrow abilities of crystallized intelligence (Gc), processing speed (Gs), and working memory (Gsm) are all related to mathematics and language achievements to varying degrees. In addition, auditory processing (Ga) and long-term retrieval (Glr) are also considered to be related to reading. Visual processing (Gv) is not related to reading and mathematics achievements, but may be related to advanced mathematical skills. In addition, more and more researchers pay attention to the role of executive function in the development of students’ academic achievements. Some test development has incorporated executive function into their test content, such as the measurement of four abilities, including planning, attention, simultaneous and subsequent processing, and test system in the cognitive evaluation. Some researchers believe that the first two tests are the measurement of students’ executive function [4].

The research on specific cognitive ability plays an important role in helping teachers understand the causes of learning difficulties of students. However, most of the current research on cognitive ability and academic achievements are isolated to investigate a certain cognitive ability or cognitive process. In addition, very little research been carried out under Chinese conditions. It is urgent to comprehensively investigate the relationship between cognitive ability and academic achievements in reading and mathematics under a complete and comprehensive CHC cognitive theoretical model. Therefore, based on the CHC theoretical model and related research on executive function, this study explored the development of crystallized intelligence (Gc), working memory (Gsm), auditory processing (Ga), long-term retrieval (Glr), processing speed (Gs), visual processing (Gv) and executive function (EF) of students with dyslexia and mathematical learning disabilities (MD) under Chinese conditions.

### 1.1. Concept Definition

In 1962, Kirk defined learning difficulties as students with normal general intelligence but long-term lagging behind, and divided learning difficulties into developmental and academic learning difficulties [5]. Academic learning disabilities mainly refer to the obstacles that students have in school learning. On this basis, Diagnostic and Statistical Manual of Mental Disorders, 5th Edition (DSM-5) divides learning disabilities into reading disabilities and arithmetic disabilities.

Dyslexia is mainly divided into two types: acquired dyslexia and developmental dyslexia. developmental dyslexia mainly refers to that students have no general audio-visual perception disorder or other neurological disorders under the same learning motivation, general intelligence and educational conditions, and their reading level is significantly lower than the normal development level of their peers [6]. DSM-5 diagnosed dyslexia as a reading level lower than the development level corresponding to their age in the standardized test of reading accuracy and understanding. In China, most scholars screen dyslexic students through necessary intelligence tests and reading ability tests. In addition, it is necessary to ensure that students’ achievements in Chinese reading are at a normal level of development [7].

Mathematics learning disability is also known as developmental dyscalculia. Its intelligence and reading level are intact [8]. Under normal educational conditions, social background and sensory skills, students are insufficient in extracting mathematical facts and executing computing procedures. At present, there is no clear diagnostic standard for mathematical learning disabilities. Most scholars in China screen students with mathematical learning disabilities through intelligence tests and necessary mathematical ability tests. In addition, students’ Chinese achievements must be kept at a normal level of development [9].

The reason for mathematical learning disabilities is that students have obvious defects in specific mathematical cognitive fields, especially in basic number processing. At present, there are four main theoretical assumptions to explain the causes of computational disabilities. One is the hypothesis of number representation defect, in which students have defects mainly in the approximate representation of larger magnitudes and the precise representation of small quantities. Studies have confirmed that compared with students with computing disabilities of the same age, there are obvious defects in the sensitivity of the approximate representation system [10] and the perception of the precise representation system [11]. The second is the number module defect hypothesis, which believes that computational disabled students have defects in the process of representing accurate quantities in the form of quantity sets, and subsequent research has also confirmed that this defect only exists in computational disabled students [12]. The third is the numerosity coding defect hypothesis, which believes that there is a defect in the internal coding system composed of brain neurons for the representation of accurate quantities by students with computational disabilities. Subsequent research confirmed this hypothesis by exploring the characteristics of defects in the number processing process of students with computational disabilities in grades 5 and 6 through sensory number and counting tasks [13]. Finally, there is the hypothesis of quantity access defect [14]. This hypothesis believes that the poor number symbol skills of students with mathematics learning disability cannot support the quantitative meaning in their acquisition of symbols. Some studies [15] conducted research on students in grades 1–4 through psychological number line task and number point task and other research paradigms, and found that the characteristics of mathematical ability defects of students in grades 1–2 in all subjects conform to this hypothesis.

In addition to dyslexia and math disorder, the comorbidity of reading disability and mathematical disability has gradually attracted scholars’ attention. Specifically, dyslexia and mathematical disorder occur simultaneously in one individual, which is similar to the concept of mathematical disorder subtype semantic memory disorder [16]. Some studies believe that the main reason for this symbiotic phenomenon may be that these obstacles are affected by the same factor [17]. The diagnosis basis for children with this type of disorder comes from the relevant studies on dyslexia and dyslexia in mathematics, which mainly means that under the conditions of normal intelligence and normal education, students have no neurological and organic injuries, and their reading ability and mathematical ability are significantly behind their peers.

### 1.2. Linking Dyslexic to Cognitive Deficits

In 1976, Hellman believed that the development of a series of complex language functions is based on auditory processing ability, which has a great impact on many academic fields of students. Among them, speech processing ability, as a special auditory processing ability, is one of the core defects of dyslexia. The main viewpoint of auditory processing disorder is that the fine processing of sound information by the auditory system over time is an important condition for the development of speech skills and a key factor affecting the development of language ability. At present, the differences in the research on the auditory processing of dyslexic students mainly focus on two aspects: on the one hand, dyslexic students have defects in the ability of fast time processing of auditory signals; for example, Martino found that compared with normal children, dyslexic children have defects in the ability to analyze different sound presentation sequences after extensive research on the time sequence judgment task of the classical experimental paradigm of auditory fast time processing ability of dyslexic children. After changing the interval time of sounds, it was found that the performance of dyslexic students in judging the order of sound presentation improved with the increase of interval time [18]. On the other hand, it is believed that the dyslexic students have the ability to process the non-temporal characteristics of auditory signals, such as frequency and amplitude changes. It is found that the dyslexic students are less sensitive to the changes of sound amplitude and frequency than the normal people. For example, some research used tasks such as speech repetition, frequency discrimination, and auditory fast time judgment to test subjects’ auditory processing ability, and found that the formation of differences in speech processing ability between dyslexic subjects and normal subjects was related to non-temporal auditory ability, but not to auditory fast time processing ability [19].

In addition, some studies divided speech processing ability into speech awareness ability, speech coding ability and speech memory ability [20], Among them, phonological awareness is the prerequisite for students’ success in reading [21]. It is also found that pronunciation plays a key role in the reading process in the Chinese environment [22]. The root of Chinese dyslexic students lies in their special defect in phonological processing ability [23]. In addition, phonological memory ability is also a key factor affecting students’ reading development. It has been found that the capacity of verbal working memory plays a crucial role in the success of reading comprehension [24]. This may be related to the process that the speech processing ability, which is regarded as the basis of reading and decoding skills, requires short-term storage of phonemes in verbal working memory.

In addition, the process of reading comprehension depends on the ability to preserve text information and integrate information in working memory. This relationship also exists in the learning process of complex Chinese environment [25]. The key role of working memory in learning has cross language consistency [26,27].

Executive function plays a key role in reading comprehension, which is composed of three independent and closely related functions: inhibition, updating and shifting [28]. In addition, the central executive system in the working memory model can be regarded as a lower level executive function [29]. According to the existing research [30], students aged 8–11 with good reading comprehension ability and those with poor reading comprehension ability have two updating tasks with an interval of one year. It is found that students with poor reading comprehension ability perform worse, receive more interference from irrelevant information, and students with poor updating ability are more likely to have reading difficulties. Further research shows that this may be due to the failure of inhibition control function, which leads students to retain more irrelevant information; that is to say, the relationship between updating ability and students’ reading comprehension is affected to some extent by inhibition control ability. Through the active interference task of 10–11 year old dyslexic children to explore the efficiency of their inhibition control, it was found that dyslexic people with poor comprehension showed specific inhibition task defects [31]. Some studies also found that the mechanism of inhibition control function in supporting reading comprehension is mainly to control irrelevant information through combing the literature [32]. In addition, this study pointed out that cognitive flexibility supports reading comprehension by integrating semantic and phonetic information and flexibly allocating attention to text features and reading strategies. Some studies assessed the relationship between the executive function and reading comprehension of 4–5 Chinese students through the classic Stroop paradigm and the span task, and found that the influence of executive function on the reading comprehension process of Chinese students exceeded the influence of vocabulary knowledge and metalinguistic awareness [33], which to a certain extent reflected the cross language consistency of the influence of executive function on reading comprehension.

In addition, some studies [34] believe that the key processing ability affecting language related achievements is processing speed ability in addition to working memory. However, the research on the relationship between processing speed and students’ reading achievements is quite contradictory. Some studies believe that this ability does not play a key role in reading achievement [35], and this view has also been confirmed by research in the Chinese environment [36].

Long term retrieval ability is significantly related to students’ reading achievements. The reading process requires students to retrieve key information from the corpus stored in their long-term memory. Some studies [37] found that the definition and measurement methods of retrieval fluency ability and rapid automatic naming ability in long-term retrieval ability almost coincide. The significant correlation between rapid automatic naming and reading [38,39] also confirms the key role of long-term extraction in the reading process. In addition, in the Chinese environment, some studies have also found that children with dyslexia perform poorly in rapid naming [40]. Some studies believe that the reason for this situation is related to the failure of the central executive system of working memory to inhibit irrelevant information during information retrieval [41].

Most Chinese characters are ideographic characters composed of semantic components and transliteration components. This complex composition in terms of vision, pronunciation and semantics puts forward higher requirements for orthography [42]. Visual spatial processing ability plays a key role in the process of Chinese students forming correct orthography knowledge. In the context of the current research on orthographic defects as the core defect of dyslexia, more and more researchers believe that dyslexia arise from deeper research on visual processing cognitive impairment, but the results are divergent. The dominant pathology theory holds that the visual defect of dyslexia is compensated by other defective abilities, which is also proved by neurophysiological research [43].

In addition, crystallized intelligence is related to the accumulation of students’ speech and vocabulary knowledge. Some studies have found that crystallized intelligence and students’ reading achievements promote each other [44]. In addition, studies on cognitive characteristics of dyslexic children have found that there are defects in their crystal intelligence [45]. Some studies have found that the defects in students’ semantic knowledge are the main reasons for their dyslexia after studying some dyslexic students who have no problems in word recognition ability [46].

### 1.3. Linking MD to Cognitive Deficits

Executive function is a key factor that affects a series of advanced cognitive skills, including mathematics. It is also a key factor that promotes students’ acquisition of mathematical skills. Different components of executive function, such as working memory [47], inhibition control [48] and cognitive flexibility [49], are key factors that affect students’ mathematical success. In a word, executive function is closely related to mathematics and dyslexia students in English environment. In the Chinese environment, some studies have found that executive function is significantly related to Chinese students’ mathematical achievements [50]. Some studies [51] have measured the executive function of 165 Chinese children aged 5–6 years by the keep track task, the Stroop task and the card sorting task, and found that working memory is the key factor affecting the mathematical achievements of Chinese students. In addition, some studies [52] measured students’ inhibition control through color stroop task and found that inhibition control was the key factor affecting students’ number concept development. For example, after measuring students’ transfer ability, it is found that transfer ability is the key factor affecting students’ mathematical achievements. Some studies [53] explored the development of transfer ability of four types of Chinese students (double excellent students, double poor students, students with math learning difficulties, and students with reading disabilities) through Wisconsin card classification task, and found that transfer function is a key factor affecting students’ math learning. Cognitive flexibility is based on working memory and inhibition control, and this ability appears only after working memory and inhibition control. The three abilities have different development trajectories. Working memory and inhibition control are the abilities that perform earlier as executive functions, which together form the basis of cognitive flexibility [54].

Students with MD have obvious defects in the central executive system of working memory, verbal working memory and visual spatial working memory. The influence of working memory on mathematics even appears in the simplest calculation process of students [55]. In English environment, students’ mathematical achievements are affected by working memory systems in general and special fields. In the Chinese environment, working memory [56] is also a key factor affecting students’ mathematical achievements. Because of language specificity, Chinese students’ verbal working memory also has a certain impact on their calculation process. For example, verbal working memory has a certain impact on Chinese students’ single digit multiplication process [57].

Students with MD in Chinese environment also have the characteristics of slow processing speed [58]. A study [59] found that processing speed is related to students’ ability to calculate and solve problems. Higher processing efficiency can increase students’ memory storage ability and improve students’ computing efficiency to a certain extent. However, some studies believe that the perception, representation and processing speed of information are crucial to the efficiency of working memory [60], which may mean that the influence of processing speed on mathematical achievements may be indirectly carried out through the way of affecting working memory.

Students with learning disabilities in mathematics are accompanied by serious defects in visual spatial addition ability. Some studies believe that students’ ability to maintain and operate visual spatial information plays a key role in students’ counting [61], computing [62], problem solving [63] and other mathematical abilities. In addition, There are research [64] findings visual reasoning ability is also a key factor affecting students’ problem solving through measuring students’ mental rotation. In the Chinese environment, some studies have found that [65] visual spatial processing disorders are significantly related to mathematical disorders. Specifically, students with MD lag behind normal children in spatial attention, spatial memory and spatial reasoning [66]. Visual spatial ability in Chinese environment can predict the development of students’ mathematical achievements to a certain extent [67].

Long term retrieval ability is also significantly related to mathematical achievement, which has also been found in Chinese environment [68,69]. Children with mathematics learning disabilities are not unable to remember correctly and retrieve facts. There may be three mechanisms that affect students’ retrieval ability [70]. One is that the representation of voice, language and voice established in long-term memory of students with mathematics learning disabilities is insufficient, which affects the development of students’ counting ability at the initial stage of mathematics learning to a certain extent. The second is that students’ ability to restrain irrelevant information entering working memory is insufficient in the process of mathematical fact retrieval. The third is that there are defects in the students’ quantity representation system, which includes an approximate number system and Precise number system. It helps students establish digital psychological representation through basic numerical processing. In fact, this may be very important in the students’ mathematical learning process, and it is the basis for students to learn basic arithmetic and learn more advanced mathematical knowledge and skills [71]. Generally speaking, the more correct memory representations that students can retrieve, the better their math scores will be. Otherwise, students may have difficulties in mathematical calculation and problem solving [72,73].

Most studies have found the correlation between crystallized intelligence and mathematical achievement. Even if it is refined to different aspects of mathematical learning process, such as mathematical calculation and problem solving, crystallized intelligence is also a key factor affecting its development [59,74]. For example, students with low mathematical achievement rely more on previous mathematical facts or knowledge than higher reasoning ability in the process of solving problems.

This study has the following specific purposes: (1) to determine whether the cognitive abilities of CHC are significantly related to the reading achievements and mathematical achievements of Chinese children. (2) explore how cognitive factors predict Chinese children’s reading and mathematics learning. (3) explore whether there are differences in the prediction of disabled Chinese children’s reading or math cognitive abilities

## 2. Research Methods

The main goal of the study was to evaluate how well children with dyslexia and mathematical disabilities performed in reading and math using their cognitive abilities. Due to the nature of the current study, which is being conducted with students in grades 1–6 in Xi’an, Shaanxi Province, China, correlational methods and comparisons between the study participants’ groups were used. Furthermore, correlational statistics have been used to discover relationships between or among variables [75]. A research tool must be thoroughly evaluated before it can be said to have excellent validation, which means that it is both reliable and valid. This design was found to be ideal for this study because it allows researchers to collect information about the prevalence and nature of learning disabilities afflicting primary school students [76].

### 2.1. Participants

By stratified sampling, 427 Chinese children aged 6 to 12 years were randomly selected from a primary school in Xi’an from grade 1–6. These samples (average age 114 months, range 67 to 148 months) are mainly used to explore the differences between the core cognitive factors affecting Chinese students’ reading and math performances.

Students with dyslexia or MD were screened from all students in grades 1–6 of a primary school in Xi’an according to the Criteria for screening dyslexia and MD in Chinese related studies [7,9,77,78,79]:

Screening criteria for dyslexia: ① In the mid-term and final examination, the scores of dyslexic children were lower than 15% of the whole class. ② The z score of dyslexic children in the reading proficiency test is more than 0.5 standard deviation which is lower than that in the math proficiency test. ③ Consistent with the evaluation of the teacher. ④ The children are at normal level in the Raven IQ test. According to the above criteria, the dyslexia group consisted of 34 children (25 males and 9 females) with an age range of 77–147 months (averaging 118 months).

Screening criteria for mathematical learning disabilities: ① In the mid-term and final examination, the scores of children with MD were lower than 15% of the whole class. ② The z score of children with MD in the math proficiency test in this study was more than 0.5 standard deviation which is lower than that in the reading proficiency test. ③ Consistent with the evaluation of the teacher. ④ The children are at normal level in the Raven IQ test. According to the above criteria, the MD group consisted of 34 children (18 males and 16 females) with an age range of 67–148 months (averaging 118 months).

In addition, the average group consisted of 34 children (20 males and 14 females) with an age range of 72–148 months (averaging 120 months). These children received scores that were within normal range of IQ. None of them had a history of learning disabilities, language impairment, emotional disturbance, and ADHD. In addition, they had normal or corrected-to-normal vision and were screened for normal hearing.

In terms of group differences, ANOVA test showed no significant age differences among the three groups of this study: F (2, 99) = 0.126. *p* > 0.05. In addition, a chisquare test indicated that the composition of the three groups of this study was balanced by gender: χ2 (2) = 3.238, *p* > 0.05. The above results prove that there is no significant difference in age and sex among the three groups in this study [80].

### 2.2. Research Materials

This study is based on the Cattell Horn Carroll (CHC) theory and related research on executive function, through reference to the Woodcock Johnson Cognitive Tests (WJ IV COG) [81] and the classic executive function tests, we developed the following measurements to test the general cognitive abilities tests:

Test 1: Crystallized Intelligence: In this study, After the word frequency statistics and analysis of Chinese characters and phrases in the Chinese textbooks compiled by the Chinese Ministry, the words and phrases are randomly selected at different levels and divided into six grades from easy to difficult, with a total of four sub tests: (1A) Vocabulary knowledge, (1B) Close Synonym, (1C) Antonyms, and (1D) Word analogy, the reliability of the subtest is 0.93, and the reliability is qualified.

Test 2: Working Memory: Short-term memory (Gsm) is tested by the Working Memory test. In this study, students’ working memories were measured by referring to the number span task of the previous classic psychological experimental paradigm [82], and the reliability coefficient of this test was 0.71.

Test 3: Spatial Relations: The spatial relationship test used in this study, which has a reliability coefficient of 0.84, This test requires students to select the correct graphic part according to the known complete graphic style, which is divided into two dimensions according to the complexity of the complete graphic composition, and students need to select 2 graphic parts for the first dimension; The second dimension is 3.

Test 4: Auditory attention: Auditory attention is a text of Auditory Processing (Ga), This test requires students to play the recording after helping them identify the target stimulus. Students need to make correct responses to the target stimulus in the recording, and the reliability coefficient of this test was 0.79.

Test 5: Long-Term Retrieval: Long-Term Retrieval is a test of Long-Term Retrieval (Glr), By sorting out the literature on the research of long-term extraction, and according to the principle of long-term retrieval test design in the relevant literature, we can enrich the presentation mode of test graphics and increase the number of graphics (such as square, triangle, trapezoid, and complex composite graphics), and increase the difficulty of visual processing, with a reliability of 0.77.

Test 6: Processing Speed: The processing speed test is conducted by crossing out the target number. Students are required to do it in two ways within the specified time, The first is to cross out the target number in front of 6, and the second is to cross out the number set with adjacent two digits and 10. The reliability coefficient of the test is 0.76.

Test 7: Executive Functions: The executive control subtest was administered in reference to the classical psychological research paradigm of the Stroop task. The test consists of two subtests: (7A) Counting Stroop Test and (7B) Color Stroop Test, with a reliability of 0.80.

Test 8: Chinese reading ability test, it mainly inspects the students’ masteries of the meaning of the material, and controls the complexity of the paragraph material by controlling the frequency of the occurrence of the strange words in the key sentences in the paragraph material, and mainly inspects the students’ reading levels, the reliability coefficient is 0.702–0.821.

Test 9: mathematics achievement level test, which includes four aspects: calculation, number perception, geometric space relationship and problem-solving ability. It mainly inspects students’ mathematical abilities; the reliability coefficient is 0.735–0.870.

### 2.3. Procedures

After stratified random sampling by grade from the overall 1–6 grades of an experimental primary school in Lianhu District, Xi’an City, Shaanxi Province, China, 427 students (with an average age of 114 months, ranging from 67 to 148 months) were obtained. These students were all enrolled in the 2020/2021 school year, respectively, for crystallized intelligence, working memory, spatial relations, auditory attention, long-term retrieval The general cognitive ability assessment tool test, including processing speed and executive function, and the mathematics and Chinese reading proficiency test for primary school students. The actual test time of the general cognitive ability assessment test is 1 to 1.5 h for each student, and the entire test period is about 6 months. Before the test, parents have obtained written consent for students to participate in the test.

Based on screening criteria determine the MD group (*n* = 34) and the dyslexia group (*n* = 34). Normal children (*n* = 34) were randomly selected as control group according to grade and sex. Participants will be assessed in the 2020/2021 academic year. The participants received seven general cognitive assessment tests, obtained the consent of parents for students’ participation before the test, and provided rewards for the participants. The data were collected by the author and three other researchers. These researchers all have educational psychology related background or special education degree. In the process of data collection, the author will update and discuss with the examiner team every day to solve the key points in test management and provide feedback.

To ensure the ethical issue, written permission from the School of Education—Research Ethics Review Committee was obtained before interacting with or gathering student data. The ideals of the schools have been adopted for use. Students were also notified and consented to the collection of data. Furthermore, it was made clear that all data would be kept confidential and utilized only to further the goals of this study.

## 3. Results

### 3.1. Correlation Analysis of Cognitive Abilities and Chinese Mathematics Achievements

The descriptive statistical results of students’ scores, averages and standard deviations in various cognitive abilities and Chinese and mathematics achievements are shown in the following Table 1.

It can be seen from Table 2 above that all cognitive abilities are significantly correlated with students’ mathematical achievements, with the absolute value of correlation between 0.111–0.473. Among all cognitive abilities, the highest correlation coefficient between executive function and students’ mathematical achievements is 0.473. Secondly, the correlation coefficient of spatial relationship ability was 0.460, and the working memory ability was 0.411.

Each cognitive ability is significantly related to students’ Chinese reading levels, with the absolute value of correlation between 0.169–0.363. Among all cognitive abilities, the correlation coefficient between crystallized intelligence and students’ reading achievements is the highest 0.363. Secondly, auditory processing was 0.337, executive function was 0.325, and working memory was 0.318.

The results of regression analysis in Table 3 show that executive function, spatial relationship and working memory are the cognitive abilities that predict students’ mathematical achievements. The three variables together explain 32.6% of the variance of students’ mathematical achievements. Among them, executive control can explain 22.3% of the variance.

The cognitive ability to predict students’ reading performances is crystallized intelligence, auditory processing and working memory. The three variables together explain 20.8% of the variance of students’ reading performances, of which crystallized intelligence can explain 13.3%.

### 3.2. Comparison of Cognitive Development among the Study Groups

It can be seen from the Table 4 that there is no significant difference in variance among the three groups, and *p*-value was higher than 0.05 for each factor (*p* > 0.05), which can be compared.

From Table 5, we can see that there are significant differences in cognitive abilities between different experimental groups. It can be seen from the above Table 6 that the MD group and the dyslexia group lag behind normal students in seven cognitive ability dimensions, among which, the dyslexia group are significantly lower than the MD group in auditory processing ability. In addition, crystallized intelligence, working memory, visual processing and processing speed are lower than the MD group but not reach a significant level. In executive function, the MD group are lower than the dyslexia group, and reach the marginal significant level.

## 4. Discussion

### 4.1. An Analysis of the Correlation and Predictive Effect of Cognitive Ability on Chinese Students’ Reading and Mathematical Achievements

In this study, there are similarities and differences in the influence of various cognitive abilities on mathematics and Chinese students’ achievements. The common factor in this study is that working memory is significantly related to the reading and mathematical achievements of Chinese students in grades 1–6, and has a high predictive effect on the reading and mathematical achievements of Chinese students, which can explain 2% of the variation in reading achievement and 3% of the variation in mathematical achievement, respectively. A large number of studies have found that working memory ability plays a key role in different aspects of students’ mathematical achievements, such as computing, problem solving, etc. [83,84]. This study has proved this by studying Chinese children. In addition, studies have found that the key to successful Chinese reading lies in the integration of orthographic knowledge in memory and morphological awareness [85]. A large number of studies have proved that working memory plays a key role in helping Chinese students store morphological awareness and orthographic knowledge [33,86].

Specifically, there are differences in the core factors that affect students’ reading and math achievements. In this study, Crystallized intelligence is significantly related to Chinese students’ reading achievement, and can explain 13% of reading achievement variation. Crystallized intelligence is the key factor in predicting Chinese students’ Chinese achievements. Crystallized intelligence represents language understanding and reasoning ability to a certain extent. Language understanding has a unique predictive effect on reading achievement, and reasoning ability is also a key factor in the development of advanced skills such as reading comprehension. The root of the influence of crystallized intelligence on reading achievement is that students’ mastery of word meaning is the key factor for successful reading. The premise for successful reading is that students must be able to understand the concept of the text they are reading, or they need to associate previous knowledge related to speech with the content of the text they are reading. The follow-up study [87] also proved that children’s ability to understand lexical texts can predict reading. The influence of crystallized intelligence on students’ reading achievement continues to increase with their age [88]. This study confirmed this by comparing the development of crystallized intelligence of junior and senior students.

In this study, spatial relationship and auditory processing are significantly related to Chinese students’ reading achievement. In particular, auditory processing can explain 5% of the variation of reading achievement, which has a high predictive effect on Chinese students’ reading achievement. The basic cognitive ability represented by spatial relationship and auditory processing ability is the core cognitive factor affecting Chinese students’ reading achievement. In the lower grades, auditory processing ability plays a leading role, which is related to the special process of Chinese character learning. Chinese learning requires students to master the phonological awareness, including syllable awareness, phonological awareness and phonemic awareness. Recognizing the phonetic structure of Chinese characters is the basis for mastering Chinese characters. This, to a certain extent, explains the reason why Chinese reading difficulties have phonetic defects [89]. Hearing and speech processing defects found in English language are special and isolated defects of students with English learning disabilities. In this alphabetic language, the phonological awareness of operating the phonological structure is more important. For example, in a study, it was found that [90] the defect in the phonological loop system in the working memory of students with reading disabilities is caused by the whole phonological processing defect, The phonological loop of speech processing and working memory is interdependent [91]. To some extent, this shows that the reading achievement of Chinese students is also affected by the advanced auditory processing ability, which is dominated by the phonological system.

As a pictographic based character, Chinese characters put forward higher requirements for students’ visual spatial cognitive process to a certain extent. The study found that [92] visual spatial attention ability is a key factor affecting Chinese students’ orthographic awareness and reading fluency. In addition, visual processing ability plays a key role in helping Chinese students identify similar Chinese characters and improving reading fluency [93]. Studies have proved that the impact of visual processing ability on reading will gradually decrease with the generation of students’ advanced reading skills and the automation of reading process [94], and the impact of visual processing ability on reading achievement will decrease with age, which is consistent with this study.

In this study, executive function is significantly related to Chinese students’ reading achievement. This study investigates the executive function of students through the color word and number Stroop test, focusing on inhibition control and cognitive flexibility. Some studies have found that inhibition control ability can help Chinese students suppress irrelevant semantic interference and correctly activate target Chinese characters, It plays a key role in achieving efficient reading [95]. In addition, in the Chinese language environment, complex orthography knowledge also requires students to flexibly extract key information from working memory storage according to different task requirements [95], which to a certain extent puts forward higher requirements for Chinese students’ cognitive flexibility. The follow-up study [96] also proved that different aspects of executive function play a key role in the reading process of Chinese children, and the results of this study are consistent with them.

Executive function is also a core cognitive factor that affects Chinese students’ mathematical achievements. In this study, executive function is significantly related to Chinese students’ mathematical achievement, and has a high predictive effect on Chinese students’ mathematical achievement, which can explain 22% of the variance of mathematical achievement. The inhibition of irrelevant information is the key to improving working memory efficiency [58]. Subsequent research on the problem solving ability of students with mathematics learning disabilities has proved this [97]. In this study, it was found that the lower grade students mainly relied on working memory, while the higher-grade students’ mathematical achievements were more susceptible to the influence of inhibition control and cognitive flexibility. The higher-grade students were required to have more efficient working memory for more complex mathematical tasks, and inhibition control played a key role in this process. In addition, in this study, spatial relationship ability is the core cognitive factor to predict Chinese students’ mathematical achievements, which is consistent with the related research on the relationship between spatial relationship ability and mathematical achievements [98].

Finally, In this study, processing speed and long-term retrieval ability are significantly related to Chinese students’ reading and mathematical achievements, but they are not the core cognitive factors that predict Chinese students’ reading and mathematical achievements. But specifically, in this study, the processing speed is highly related to the reading and mathematical achievements of Chinese students, which may imply that processing speed has an indirect impact on Chinese students’ math and reading achievements. For example, it is also believed that [99] dyslexia is more likely to be affected by processing speed when manipulating memory in the process of speech processing, which leads to students’ dyslexia. In fact, this is to affirm that processing speed is an insufficient condition for the backward development of reading, which has also been proved by research in the Chinese environment [36].

### 4.2. An Analysis of Cognitive Differences between Chinese Children with Reading Difficulties, MD and Normal Students

This study is consistent with previous studies. In this study, compared with normal students, the crystallized intelligence, working memory, spatial relationship, auditory processing, processing speed, long-term retrieval and executive function of the dyslexia group and the MD group are significantly lower than those of normal students. In addition, compared with the MD group, the dyslexia group have significant auditory processing defects, crystallized intelligence, working memory, visual processing and processing speed abilities are also low, but they do not reach a significant level. Compared with the students with reading difficulties, the MD group have significant executive dysfunction. In addition, the long-term retrieval ability is also low, but it does not reach a significant level.

Among them, Chinese dyslexic students have more obvious auditory processing disorder, which is consistent with some studies in English environment. Phonetic processing ability is the core defect of children with dyslexic. In the process of learning Chinese, some studies have found that, for example, dyslexia is not sensitive to side voice cues [100], which leads to more semantic errors of students in the reading process. Fast naming ability has also been found to be a key factor affecting students’ reading process. Some studies have found that in the Chinese environment, fast naming ability includes three parts: speech processing, Chinese recognition, and pronunciation actions [101], It proves that phonological processing ability has a key influence on Chinese reading process.

In this study, Chinese dyslexic children have worse spatial relationship ability, which is different from English environment related research [102], which may be related to Chinese language specificity. In the Chinese environment, the researcher [103] found that visual spatial attention ability was significantly related to Chinese character processing skills such as orthographic awareness and spelling ability of Chinese characters to some extent. In addition, some studies [104,105] found that the visual spatial working memory ability of Chinese dyslexic students is significantly lower than that of normal children. Some studies [106] pointed out that individuals can use the resources in the voice loop to process visual spatial information, thus relieving the pressure of visual processing. However, in this study, it was found that children with dyslexia have significant defects in working memory related to speech, which makes them unable to share the pressure for visuospatial power. The main system hypothesis of connectionism holds that the connection among vision, pronunciation and semantics determines students’ reading processing. This hypothesis holds that the root cause of reading disorder is the impairment of students’ basic cognitive system, which is proved to some extent by this study.

In this study, compared with the MD group, the working memory and processing speed of the students with reading difficulties were worse, but did not reach a significant level. It has been found that [40] Chinese children with dyslexia also have extensive working memory related defects. The root of working memory defects of Chinese dyslexia students may lie in the defects of the phonological loop in working memory [36]. The phonological loop and phonological processing ability in working memory may have common basic abilities [90,91]. The phonological processing ability of dyslexia students in this study may also have obvious defects, both of them are the common cause of Chinese students’ reading disorder. A large number of studies believe that the impact of processing speed on students’ academic achievements is more indirect, and the speed at which information is introduced into working memory and processed has a great impact on working memory efficiency [107]. In this study, the correlation between processing speed and working memory is also significant. The complexity of orthography knowledge of Chinese characters puts forward higher memory requirements for students to a certain extent, so processing speed may also affect the reading process of Chinese students through this indirect way.

In this study, in terms of crystallized intelligence, students with reading difficulties and MD are significantly lower than normal students, which is consistent with previous studies, and the dyslexia group are worse than the MD group, but there is no significant difference. A large number of studies have proved that [108] crystallized intelligence is one of the core cognitive factors that predict all academic fields of students, such as reading and mathematics. This study confirms that the reading process of Chinese children is also affected by crystallized intelligence. This proves to some extent that [109] crystallized intelligence has a cross linguistic effect on reading achievement, and is not affected by ethnic language differences. In addition, in this study, the MD group also have some defects in crystallized intelligence, which is consistent with students with mathematics learning difficulties in English environment [110].

In this study, the executive function of the dyslexia group and the MD group is significantly lower than that of normal students, and that of the MD group is lower than that of the dyslexia group, reaching a marginal significant level. The complexity of the knowledge of Chinese orthography puts forward higher requirements for students’ executive function. In the relevant research on Chinese children, it is found that working memory, inhibition control and cognitive flexibility play a key role in affecting students’ reading process [111,112]. In addition, in this study, the MD group also have extensive executive function defects. Some studies have found that [113] has a greater impact on students’ mathematical disabilities than students’ linguistic achievements, especially in primary school [114]. Different aspects of executive function can predict the development of students’ mathematical achievements. Mathematical tasks gradually require students to have higher level skills including executive function with the increase of students’ grades [115], which to some extent explains that students with mathematical disabilities have worse executive function.

In terms of long-term retrieval, the long-term retrieval ability of the dyslexia group and the MD group is significantly lower than that of normal students, but there is no significant difference between the dyslexia group and the MD group. Some studies believe that the process of long-term retrieval is an automated process [116,117]. This automated process is manifested in the need for students to quickly and smoothly extract relevant information from long-term memory to help them understand the text [118,119]. Therefore, a large number of studies believe that long-term retrieval ability is significantly related to multiple fluency abilities, which are expressed in the reading process as word fluency, etc., and in the mathematics field as thinking fluency, etc., Subsequent research [120] proved that long-term extraction ability plays a key role in students’ mathematical achievements such as problem solving.

### 4.3. Strength and Limitations

This research is the first attempt to explore the cognitive development of Chinese students (Dyslexia, math difficulties, and normal children) under the framework of CHC theory. However, some aspects of this research should be improved. One is that the content of cognitive ability measurement for students is not comprehensive and detailed enough, which may lead to certain limitations of the results of this research, for example, in the research on visual memory, it is found that dyslexics have certain visual advantages [121], but in some studies [122], it is found that as long as the visual memory interval of dyslexic students is increased, dyslexic students will lag behind normal development students. Secondly, the sample size in this study is small, and a larger sample size is needed to confirm the findings of this study. Third, in this study, it seems necessary to divide the control group into two groups, the age matched control group and the ability matched control group [123], to compare the differences between students with reading disabilities, students with math disabilities and their peers. In addition, the development of students’ cognitive function is evaluated from the perspective of brain function through resting state functional magnetic resonance imaging (fMRI) counting. For example, in a study on the process of Chinese reading, students need to activate the left fusiform gyrus in the process of character shape processing [124], while in the process of Chinese reading, shape sound processing is related to the left inferior frontal gyrus and insular lobe [125], which to some extent indicates that students’ cognitive process is the manifestation of brain function, The cognitive development of students can also be assessed by exploring the level of brain activity in specific cognitive processes of students, which needs further research.

This study hopes to measure the cognitive development of students, so as to find out and master the reasons for the backward learning of students with reading disabilities and students with mathematics learning difficulties in a timely manner, so as to provide teachers with professional and personalized intervention plans, which is the premise for scientific intervention of students.

## 5. Conclusions

1. Chinese children with dyslexic have extensive auditory processing defects; Children with MD have worse executive function.
2. The basic cognitive abilities, such as spatial relationship ability and auditory processing ability, are crucial to the reading achievement of low-grade Chinese children. Under the influence of Chinese language specificity, spatial relationship ability is the key factor to predict the reading achievements of Chinese children in the lower grades of primary schools.
3. The core cognitive factors for predicting Chinese dyslexia students are crystallized intelligence, auditory processing and working memory. Among them, the influence of crystallized intelligence on Chinese students’ reading achievements increases with their age.
4. Executive function, spatial relationship and working memory are the core cognitive factors that predict Chinese children’s mathematical achievements.
5. The cognitive status of children with Chinese reading difficulties and MD is significantly lower than that of normal students.

## Figures and Tables

**Table 1:** Scores of Chinese students in grades 1 to 6 in Chinese, mathematics and cognitive abilities.

	M	SD	Min	Max
Gc	122.78	37.48	16.00	175.00
Gsm	7.17	2.72	1.00	16.00
Gv	60.09	7.57	23.00	76.00
Ga	204.70	13.87	128.00	218.00
Glr	92.61	12.88	2.00	107.00
Gs	66.84	13.42	5.00	115.00
Ef	95.25	17.33	35.00	126.00
M	72.24	22.19	12.00	100.00
R	67.77	20.67	12.00	99.00

Notes: M = Mean, SD = Standard Deviation, Gc = Crystal Intelligence, Gsm = Short-Term Memory, Gv = Visual Processing, Ga = Auditory Processing, Glr = Long-Term Retrieval, Gs = Processing Speed, EF = Executive Functions, R = reading achievements, M = math achievements.

**Table 2:** The cognitive abilities of Chinese students in grades 1 to 6 are related to their Chinese mathematics scores.

	Gc	Gsm	Gv	Ga	Glr	Gs	Ef	M	R
Gc	1								
Gsm	0.497 **	1							
Gv	0.540 **	0.338 **	1						
Ga	0.318 **	0.200 **	0.254 **	1					
Glr	0.236 **	0.166 **	0.158 **	0.373 **	1				
Gs	0.451 **	0.312 **	0.448 **	0.269 **	0.199 **	1			
Ef	0.603 **	0.470 **	0.469 **	0.277 **	0.214 **	0.568 **	1		
M	0.405 **	0.411 **	0.460 **	0.178 **	0.111 *	0.382 **	0.473 **	1	
R	0.363 **	0.318 **	0.274 **	0.337 **	0.228 **	0.205 **	0.325 **	0.169 **	1

Note: (**) *p* ≤ 0.01, (*) *p* ≤ 0.05.

**Table 3:** Effects of cognitive abilities on Mathematics and Chinese students scores in math and reading (grades 1 to 6).

Subect	Variable	*R* ^2^	Δ*R*^2^	*B*	*SE*	*β*	*t*	*p*
Math Reading	Ef	0.223	0.221	00.32	0.062	0.25	50.126 ***	0.000
	Gv	0.072	0.071	0.805	0.134	0.274	50.984 ***	0.000
	Gsm	0.031	0.029	10.639	0.374	0.201	40.38 ***	0.000
	Gc	0.133	0.131	0.114	0.028	0.206	30.994 ***	0.000
	Ga	0.054	0.052	0.351	0.068	0.236	50.158 ***	0.000
	Gsm	0.021	0.019	10.279	0.38	0.168	30.365 ***	0.001

Note: (***) *p* ≤ 0.001.

**Table 4:** One-way ANOVA tests on each of the seven CHC factors according to the study groups.

	SD			*F*	*p*
Variable	1.0 (*n* = 34)	2.0 (*n* = 34)	3.0 (*n* = 34)		
Gc	34.719	40.134	33.980	0.503	0.606
Gsm	3.134	3.111	2.719	1.267	0.286
Gv	10.030	11.224	12.781	2.026	0.137
Ga	14.923	17.648	14.046	2.066	0.132
Glr	14.513	14.959	18.939	0.711	0.494
Gs	12.644	16.088	15.270	0.93	0.398
EF	19.428	22.480	22.872	2.264	0.109

**Table 5:** The effect of CHC factors on the study groups (Normal children, Dyslexia, Math difficulties).

Variable	NC		D		MD				
	M	SD	M	SD	M	SD	*F*	*p*	*r*
Gc	134.882	34.719	110.676	40.134	110.765	33.980	4.999 **	0.009	0.292
Gsm	8.382	3.134	6.676	3.111	6.941	2.719	3.197 *	0.045	0.254
Gv	60.206	10.030	53.294	11.224	54.176	12.781	3.702 *	0.028	0.273
Ga	208.294	14.923	192.441	17.648	205.824	14.046	10.145 ***	0.000	0.453
Glr	96.088	14.513	87.765	14.959	87.176	18.939	3.195 *	0.045	0.254
Gs	74.941	12.644	58.206	16.088	64.441	15.270	11.193 ***	0.000	0.476
EF	100.118	19.428	87.412	22.480	77.559	22.872	9.583 ***	0.000	0.440

Note: (***) *p* ≤ 0.001, (**) *p* ≤ 0.01, (*) *p* ≤ 0.05; M = Mean, SD = Standard Deviation, F = Observed F Value, *p* = Significance Level. NC = Normal Children, r = Effect Size = Dyslexia, MD = Math Difficulties. Gc = Crystal Intelligence, Gsm = Short-Term Memory, Gv = Visual Processing, Ga = Auditory Processing = Long-Term Retrieval, Gs = Processing Speed, EF = Executive Functions.

**Table 6:** The results of multiple comparisons after ANOVA among the study groups in grades 1 to 6.

	Variable (I)	Variable (J)	Mean (I)	Mean (J)	Difference (I–J)	*p*
Gc	1.0	2.0	134.882	110.676	24.206 **	0.010
	1.0	3.0	134.882	115.412	19.471 *	0.023
	2.0	3.0	110.676	115.412	−4.735	0.602
Gsm	1.0	2.0	8.382	6.676	1.706 *	0.028
	1.0	3.0	8.382	6.941	1.441 *	0.047
	2.0	3.0	6.676	6.941	−0.265	0.710
Gv	1.0	2.0	60.206	53.294	6.912 **	0.009
	1.0	3.0	60.206	54.176	6.029 *	0.034
	2.0	3.0	53.294	54.176	−0.882	0.763
Ga	1.0	2.0	208.294	192.441	15.853 ***	0.000
	1.0	3.0	208.294	205.824	2.471	0.485
	2.0	3.0	192.441	205.824	−13.382 ***	0.001
Glr	1.0	2.0	96.088	87.765	8.324 *	0.023
	1.0	3.0	96.088	87.176	8.912 *	0.033
	2.0	3.0	87.765	87.176	0.588	0.887
Gs	1.0	2.0	74.941	58.206	16.735 ***	0.000
	1.0	3.0	74.941	64.441	10.5 **	0.003
	2.0	3.0	58.206	64.441	−6.235	0.106
Ef	1.0	2.0	100.118	87.412	12.706 *	0.015
	1.0	3.0	100.118	77.176	22.941 ***	0.000
	2.0	3.0	87.412	77.176	10.235 *	0.067

Note: (***) *p* ≤ 0.001, (**) *p* ≤ 0.01, (*) *p* ≤ 0.05.

## Data Availability

The data that has been used is confidential.
